# A Text-Book of Alkaloidal Therapeutics

**Published:** 1904-11

**Authors:** 


### Books and Magazines.

A Text-Book of Alkaloidal Therapeutics.—Being a Condensed Resumé of all Available Literature on the Subject of the Active Principles Added to the Personal Experience of the Authors. By W. F. Waugh, M. D., and W. C. Abbott, M. D., Chicago: The Clinic Publishing Co. 1904. Price, $2.50.

This work is dedicated to those who believe in "the smallest possible quantity of the best obtainable means to produce a desire in therapeutic results." Drs. Waugh and Abbott are pioneers in this new and advanced field of therapeutics, and they give the profession, in this neat hand-book, a resumé of their research and investigation. It has been painstaking and thorough, and they present here a mass of very valuable information thoroughly digested. The reduction of our therapeutics to the active principle of drugs, eliminating the useless and crude parts, constitutes one of the most valuable advances in the science of medicine and the art of pharmacy within a century. No more bulky and nauseating mixtures need be prescribed, and put up by that back number, the prescription druggist. It is the part of wisdom, expediency, economy, safety and certainty to adopt the methods of dispensing one's own medicines, and the alkaloids represent the essence of what you desire to give your patient. See editorial in our September number. Every progressive doctor should investigate alkaloidal therapeutics. D.

